# Prevalence of Congenital Malaria in Minna, North Central Nigeria

**DOI:** 10.1155/2012/274142

**Published:** 2011-08-14

**Authors:** Innocent Chukwuemeka James Omalu, Charles Mgbemena, Amaka Mgbemena, Victoria Ayanwale, Israel Kayode Olayemi, Adeniran Lateef, Victoria I. Chukwuemeka

**Affiliations:** ^1^Department of Biological Sciences, Federal University of Technology, Minna, Nigeria; ^2^Dentistry Department, Niger State General Hospital, Minna 900002, Nigeria; ^3^Department of Biochemistry/Physiology, University of Abuja, FCT, Nigeria

## Abstract

The study was designed to determine the true prevalence of congenital, cord, and placental malaria in General Hospital Minna, North Central Nigeria. Peripheral blood smears of near-term pregnant women, as well as the placental, cord, and peripheral blood smears of their newborn babies, were examined for malaria parasites, using the Giemsa staining technique. Out of 152 pregnant women screened, 21 (13.82%) of them were infected with malaria parasites. Of the 152 new born babies, 4 (2.63%) showed positive peripheral parasitaemia. Placental parasitaemia was 7/152 (4.61%), while cord blood parasitaemia was 9/152 (5.92%). There were strong associations between peripheral and cord malaria parasitaemia and congenital malaria (*P* < 0.05). *Plasmodium falciparum* occurred in all, and none had mixed infection. The average birth weights of the babies delivered of nonmalarious pregnant women were higher than those delivered by malarious pregnant women, though not significant (*P* > 0.05). Malaria parasitaemia occurred more frequently in primigravidae than multigravidae.

## 1. Introduction

Congenital malaria was first described in 1876 [1]. It can be acquired by transmission of parasites from mother to child during pregnancy or perinatally during labour [2].

Congenital malaria has been documented for many years, but it was previously thought to be uncommon especially in indigenous populations; more recent studies, however, suggest that incidence has increased, and values between 0.30 to 33.00% have been observed from both endemic and nonendemic areas [3].

Malaria and pregnancy are generally believed to be mutually aggravating conditions. The pathological changes due to malaria and the physiological changes associated with pregnancy have a synergistic effect on the course of each other [4].

Pregnancy exacerbates malaria through a nonspecific hormone-dependant depression of the immune system; protective antiplasmodial activity is suppressed at pregnancy [5].

The following hypotheses to explain the altered immunity associated with pregnancy were offered.

Reduced lymphoproliferative response sustained by elevated levels of serum cortisol, loss of cell-mediated immunity in the mother, the presence of placenta, a new organ in the primigravidae, allows the parasite to bypass the existing host immunity, or allows placenta-specific phenotypes of *P. falciparum* to multiply; pregnant women display a bias towards type-2 cytokines and are therefore susceptible to diseases requiring type-1 responses for protection like TB, malaria, and *P. falciparum *has the unique ability of cytoadhesion; chondroitin sulfate A and hyaluronic acid are the adhesion molecules for parasite attachment to placental cells [4].

In pregnancies complicated by malaria, both fetal growth retardation and preterm birth contribute to low birth weight [6]. Malaria control still remains the foremost public health challenge in Africa, and world malaria report, which indicated that Nigeria accounts for a quarter of all malaria cases in the 45 malaria endemic countries in Africa, clearly showed the enormity of the socioeconomic and health burdens of the disease in the country, even in the face of dearth of information on the epidemiology of the disease. This, thus, informed this study on the prevalence of congenital malaria in Minna, an often neglected but germane aspect of the epidemiology of malaria in endemic communities [7].

## 2. Materials and Methods

### 2.1. Study Site and Population

The study was carried out at General Hospital Minna, North Central Nigeria. Minna, the capital of Niger State, Nigeria, is located within longitude 6°33′E and latitude 9°37′N, covering a land area of 88 km^2^ with a population of 1.2 million. Minna has a tropical climate with mean annual temperature, relative humidity, and rainfall of 30.20°, 61.00%, and 1334.00 cm, respectively. The climate presents two distinct seasons: a rainy season (April–October) and dry season (November–March). Minna is an endemic area for malaria.

The study was conducted from October 2010 to February 2011. The subjects were near-term (close to delivery) pregnant women who were delivered of their babies at the hospital. Sample sizes were determined from the number of pregnant women that attended antenatal care during the period of study. The nonpregnant women served as control to help compare malaria prevalence in pregnancy only. The pregnant women did not take any chemoprophylaxis and did not use mosquito treated nets for protection. The HIV status of the subjects were not determined.

### 2.2. Ethical Considerations

All work was performed according to the guidelines for human experimentations in clinical research stated by the Federal Ministry of Health of Nigeria. This study was approved by the ethical committee of General Hospital Minna, Nigeria. All women gave oral informed consent.

### 2.3. Sample Collections

Blood samples were obtained from the peripheral blood of 152 near-term pregnant women using sterile syringes. After delivery, blood was collected from the placentae by placental biopsies. The placentae were incised between the maternal and foetal surface and a small quantity of blood pipette out from the intervillous with a sterile syringe. Cord blood was collected immediately after separation of placenta, and peripheral blood of neonates was collected by heel pricking using a sterile blood lancet on clean glass slides. Labelled ethylene-diamine tetraacetic acid (EDTA) bottles were used in collecting blood samples for parasitological examinations. Blood collections were made possible with the assistance of midwives on duty during child delivery. Blood samples were also collected from 100 nonpregnant women. 

The ages, types of birth, and weights of newborn babies were recorded.

### 2.4. Parasitological Examination

Thick and thin blood films were stained with 10% Giemsa and read for malaria parasites by two trained microscopists following standard quality control procedure. Parasitaemia was expressed as the number of asexual forms of *P. falciparum *per microlitre; a result was considered negative after a reading of 1000 leucocytes in the microscope (×1000). Parasitaemia was graded as low (1–999/*μ*L), moderate (1000–9999/*μ*L), and high (>10000/*μ*L). Transplacental passage of *Plasmodium falciparum* was confirmed by detection of malaria parasite within 7 days of birth.

### 2.5. Statistical Analysis

All data were analysed using SPSS version 10.1 for windows. Descriptive statistics were computed for all relevant data. Chi square analysis was used to compare proportions within and among groups, for statistical significance.

## 3. Results

The prevalence of malaria infection in blood smears of near-term pregnant mothers, their newborns, cords, placentae, and nonpregnant women was shown in Table 1. Out of a total of 152 near-term pregnant women screened, 21 (13.82%) had parasite in their peripheral blood. Among 100 nonpregnant women screened, 13 (13.00%) had peripheral malaria parasite. Infection rate was higher in pregnant women but not significant (*P* > 0.05) using the chi-square. The inclusion of nonpregnant women helped to compare the prevalence of malaria. Also, there was no significant difference in infection rates between the primigravidae (15.79%) and the multigravidae (11.84%), (*P* > 0.05). Of the 152 newborn babies screened, 4 (2.63%) had positive peripheral parasitaemia. Examination of the 152 placentae gave a prevalence of 4.63% (7) for malaria parasite, out of which 4 placentae were for the 4 babies with positive peripheral parasitaemia and known showed any clinical symptom. The only malaria parasite encountered in this study was the *Plasmodium falciparum*.

The prevalence of cord blood parasitaemia was 5.92% (9/152), 5 of which came from multigravidae (6.58%) and 4 from primigravidae (5.26%). The average parasite density was 1,415 p/uL for pregnant women which was significantly (*P* < 0.05) higher than the 411 p/uL recorded for nonpregnant women. Average parasite density for newborns was 128 p/uL, slightly lower than that for the cords which was 196 p/uL; however, both were significantly lower than parasite density recorded in the placentae (mean = 689 p/*μ*L). Parasite density was significantly (*P* < 0.05) higher in neonates from primigravid (1281/uL) mothers than multigravids (134/uL) (Table 2). Parasite stages encountered were dominated by trophozoites (64.50%), distantly followed by gametocytes (26.80%) in pregnant women; the same trend was observed for nonpregnant women, placenta, cord, and neonates (Table 3).

The 4 parasitized newborns all had birth weights above 3 kg. On the average, however, the birth weights of babies from parasitized mothers were lower than those from nonparasitized mothers, albeit, the difference was not significant (*P* > 0.05). Also, newborns from primigravid mothers weighed averagely less than those from multigravid mothers; again the difference was not significant (Table 4).

## 4. Discussion

Many researchers have reported high prevalence of malaria in pregnancy in different parts of Nigeria, ranging from 19.70% to 72.00% [8–11]. The prevalence of malaria in pregnant women in this study was 14%; though consistent with the reported Nigerian situation, the relatively low percentage could be due to the fact that the study was carried out during the dry season, a period of low mosquito density and, perhaps, low level malaria transmission rates. This observation supports the position that in areas of malaria endemicity, pregnancy is associated with increased susceptibility to malaria, arising from pregnancy-induced altered immunity [6], Immunosuppression from raised serum cortisol, loss of cell-mediated immunity, effects of a new organ, the placenta, and loss of type-1 cytokine responses [4].

The prevalence of malaria in nonpregnant controls in this study was 13%, although this was lower than the pregnant subjects; the difference was not significant. The inclusion of nonpregnant women in the study allows comparison of malaria prevalence in pregnancy with nonpregnant female subjects. Parasite densities for pregnant women were comparatively higher than that of nonpregnant women. This is consistent with the findings of Agomo et al. [8], in a study done in Lagos, Nigeria. Although none of the pregnant women screened took malaria chemoprophylaxis during pregnancy, nor used any form of mosquito nets, the parasite density figures were low. However, one may conclude that this may have to do with the period of the study, October to February, that is, early to mid-dry season. A season that coincides with decreasing densities of female *Anopheles* species and therefore reduced inoculation with parasites. 

Low parasites densities were also recorded for the placentae, cords, and peripheral blood of newborns. 

Each placentae of the 4 parasitaemia neonates in this study also had positive parasitaemia. This agrees with the position of Chedraui et al. [6], that placental infection is a prerequisite for, but does not predict, congenital malaria. A strong correlation between placental and congenital parasitaemia has been shown [12].

However, an increasing trend in prevalence of congenital malaria has been reported recently. In a multicentre study done at Ibadan a prevalence of 5.10% was reported in University College Hospital [13]. A prevalence of 46.70% was reported in a study of 120 newborn babies at Ile-Ife, Southwestern Nigeria [14]. Also a prevalence of 13.00% was reported among 546 in-born neonates at Calabar Teaching Hospital [15]. These findings represent a new trend since parasitaemia in peripheral blood of newborns was considered rare in highly endemic areas.

Infection with *Plasmodium falciparum* was the only one encountered in this study. This agrees with a similar study done in Libreville, Gabon by Bouyou-Akotet et al. [16], wherein only *Plasmodium falciparum* was encountered. It also agrees with widely accepted view that *Plasmodium falciparum* is the predominant species in Nigeria [16]. The predominant forms of this parasite seen were the trophozoites (ring stages).

The only stillbirth encountered in this study resulted from foetal distress. The mother of the baby was negative for malaria parasites (nonmalarious).

On the average, the birth weights of babies from parasitaemia mothers were lower than those of babies from nonparasitaemia mothers, but not significantly so. This disagrees with the widely held view that babies with parasitized placentae often have low birth weight. It also contradicts the findings of Adebami et al. [17], who stated that babies of mothers with parasitized placentae have mean birth weight significantly lower than babies of mothers with nonparasitized placentae. The generally low parasite densities found in this study may explain the minimal effect on birth weight, as observed by Opara [2] who recorded parasitized neonate twins with normal birth weights. Primigravidae are more susceptible to malaria infection than multigravidae in endemic areas [18]. The findings of this study supports this position, albeit, the difference in prevalence was not significant.

Congenital malaria was previously thought to be rare, especially, in areas of malaria endemicity. Though this study appears not to support this view, an increasing prevalence of congenital malaria among newborns in other areas of malaria endemicity has been observed.

##  Conflict of Interests

The authors declare that there is no conflict of interests.

## Figures and Tables

**Table 1:** Prevalence of *Plasmodium falciparum* in blood samples of different groups of women and tissues at General Hospital Minna, North Central Nigeria.

Source of blood	Overall			Primigravidae			Multigravidae		
	No examd	No positive	Infect rate (%)	No positive	Infect rate (%)	No examd	No positive	Infect rate (%)
Near-term Pregnant women	152	21	13.82	76	12	15.79	76	9	11.84
Nonpregn women	100	13	13.00	—	—	—	—	—	—
Neonate	152	4	2.63	—	2	—	—	2	—
Placenta	152	7	4.61	76	3	3.95	76	4	5.26
Cord	152	9	5.92	76	4	5.26	76	5	6.58

Total	708	54	7.63	226	21	9.29	228	20	8.77

**Table 2:** Average parasite densities (Parasites/uL) of blood of different groups of women and tissues at General Hospital Minna, North central Nigeria.

Parasite densities	Pregnant women	Nonpregnant women	Newborn	Placenta	Cord	PG	MG
Parasites/uL	1415	411	128	689	196	1281	134

PG: Neonate with primigravid mothers,

MG: Neonate with multigravid mothers.

**Table 3:** Erythrocytic stages of *Plasmodium falciparum* in blood samples of different groups of women and tissues at General Hospital Minna, North central Nigeria.

Source of blood	No positive	TR		GM		SCH		TR & SCH		TR & GM		TR & GM & SCH		Total
		No	%	No	%	No	%	No	%	No	%	No	%	
Pregnant women	21	60	64.50	25	26.80	—	—	—	—	8	8.60	—	—	93
Nonpreg women	13	15	46.80	12	37.50	—	—	—	—	5	15.60	—	—	32
Newborn	4	5	45.50	5	45.50	—	—	—	—	1	9.00	—	—	11
Placenta	7	20	62.50	10	31.20	—	—	—	—	2	6.30	—	—	32
Cord	9	12	36.30	10	30.30	—	—	—	—	3	9.00	—	—	33

Total	54	112	62							19				201

TR: trophozoites, GM: gametocytes, SCH: schizonts.

**Table 4:** Mean (±SD) birthweights (kg) of neonate of pregnant women at General Hospital Minna, North Central Nigeria.

Pregnant women	Primigravidae		Multigravidae	
	Male	Female	Male	Female
Positive pregnant women	3.03 ± 0.5	2.83 ± 0.44	3.18 ± 0.31	2.83 ± 0.45
Negative pregnant women	3.01 ± 0.52	2.89 ± 0.25	3.31 ± 0.14	2.94 ± 0.14
